# Burden of Herpes Zoster in Individuals With Chronic Conditions in the Republic of Korea: A Nationwide Population-Based Database Study

**DOI:** 10.1093/ofid/ofae535

**Published:** 2024-09-18

**Authors:** Jing Chen, Ju-Young Shin, Sungho Bea, Byong Duk Ye, Dong-Gun Lee, Hyungwoo Kim, Won Suk Choi, Sumitra Shantakumar

**Affiliations:** Value Evidence and Outcomes (GCI), GSK, Singapore; School of Pharmacy, Sungkyunkwan University, Seoul, Republic of Korea; Department of Biohealth Regulatory Science, Sungkyunkwan University, Suwon, Republic of Korea; Department of Clinical Research Design and Evaluation, Samsung Advanced Institute for Health Sciences and Technology, Sungkyunkwan University, Seoul, Republic of Korea; School of Pharmacy, Sungkyunkwan University, Seoul, Republic of Korea; Division of Pharmacoepidemiology and Pharmacoeconomics, Department of Medicine, Brigham and Women’s Hospital and Harvard Medical School, Boston, Massachusetts, USA; Department of Gastroenterology and Inflammatory Bowel Disease Center, Asan Medical Center, University of Ulsan College of Medicine, Seoul, Republic of Korea; Division of Infectious Diseases, Department of Internal Medicine, College of Medicine, The Catholic University of Korea, Seoul, Republic of Korea; Global Medical Affairs Early Vaccines, GSK, Rockville, Maryland, USA; Department of Infectious Diseases, Department of Internal Medicine, Korea University College of Medicine, Seoul, Republic of Korea; Value Evidence and Outcomes (GCI), GSK, Singapore

**Keywords:** chronic conditions, herpes zoster, incidence, older adult, Republic of Korea

## Abstract

**Background:**

Chronic conditions (CCs) may increase the risk of herpes zoster (HZ) infection, leading to a greater healthcare burden in these individuals compared to those without CCs. It is therefore clinically important to quantify HZ disease burden in individuals with and without CCs, given the rapidly aging population in the Republic of Korea (ROK).

**Methods:**

This retrospective cohort study examines the trends in incidence rates (IRs) and incidence rate ratios (IRRs) in individuals aged ≥18 years with CCs, using the National Health Insurance Service National Sample Cohort (NHIS-NSC) database from 2010 to 2019. These patients were stratified by age group, sex, HZ complications, and CCs. The annual average number of HZ patients, IRs, and IRRs were calculated for individuals with and without CCs.

**Results:**

In total, 729 347 patients with HZ were eligible for the study. HZ IRs were highest in patients with diabetes, followed by chronic obstructive pulmonary disease, chronic kidney disease, asthma, and chronic liver disease, with HZ IRRs following a similar trend. Overall, HZ IRs generally increased with age, typically peaking at 60–64 or 65–69 years, and were similar for females and males. HZ IRs were highest among patients without complications, followed by HZ with other, cutaneous, ocular, and neurologic complications across all CCs. For each of the CCs, HZ IRs were consistently higher than those of the non-CC population regardless of sex.

**Conclusions:**

The findings of this study reiterate the importance of HZ prevention for healthy aging, especially for CC populations at increased risk of HZ in the ROK.

## Teaser

We estimated herpes zoster (HZ) incidence rates (IRs) in individuals ≥18 years with chronic conditions in the Republic of Korea. In 2010–2019, HZ IRs generally increased with age and were ∼40% higher in patients with chronic conditions than without.

## Graphical Abstract

**Graphical Abstract:**
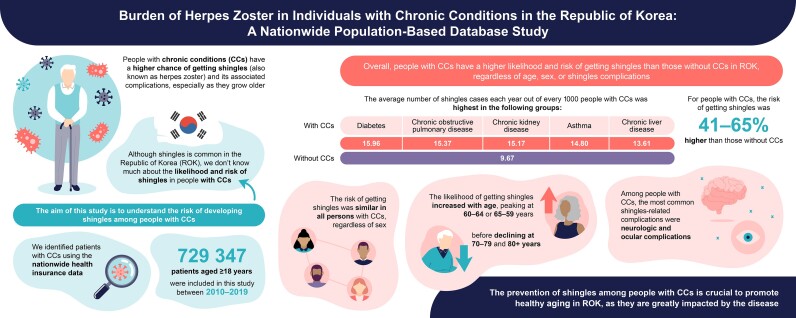

Herpes zoster (HZ) is an infectious disease caused by the reactivation of latent varicella zoster virus (VZV) and presents as a generalized, painful, pruritic, blistering skin rash [1–3]. After the initial rash has resolved, postherpetic neuralgia (PHN), a chronic and severe pain that can persist for months or even years, may follow [4, 5]. Approximately 1 in 3 individuals will experience HZ in their lifetime, and those with HZ face a 10%–18% risk of developing PHN [6].

Although the reasons for the reactivation of VZV are not fully understood, waning cell-mediated immunity resulting from factors such as advanced age, concomitant conditions, and the use of immunosuppressive medication have been identified as potential risk factors for HZ [6–8]. Despite this, approximately 90% of HZ cases occur in immunocompetent individuals, and the specific risk factors for HZ in this population remain unclear [9–11]. Chronic conditions (CCs), which have been increasing in prevalence, have also been suggested as potential causes for VZV reactivation, as the processes involved in CCs influence the functioning of the immune system [11, 12]. Several studies have reported that individuals with CCs are at higher risk of HZ infection [13–21] and may, therefore, have a greater HZ-associated healthcare burden compared to those without CCs [22–24].

Potential healthcare challenges may arise from the rapidly aging population in the Republic of Korea (ROK), as aging leads to multiple CCs, frailty, and functional decline in older adults [25]. In 2020, the average number of CCs in older adults aged ≥65 years in the ROK was 1.9, with 84% of the population having ≥1 CC [25]. Meanwhile, HZ cases in the ROK are also on the rise, as reported by a recent study based on the National Health Information Database (NHID), which covers the entire Korean population of approximately 50 million [7].

Given that patients with CCs are at a higher risk of HZ compared to their non-CC counterparts, it is clinically important to quantify the disease burden of HZ among these populations in the ROK. Therefore, this study sought to examine the trends in the incidence rates (IRs) and incidence rate ratios (IRRs) of HZ in individuals aged ≥18 years with chronic conditions between 2010 and 2019 to provide insights into the burden of HZ in this population in the ROK.

## METHODS

### Study Design and Population

A retrospective cohort study was conducted in individuals aged ≥18 years with medical records of CCs during the study period using the National Health Insurance Service National Sample Cohort (NHIS-NSC) database 2.2 from 2010 to 2019. The NHIS is a single-insurer public health insurance system covering the majority of ROK citizens and residents, which requires those insured to pay income-based premiums. Healthcare providers are reimbursed by NHIS based on billing records, who typically bill their services using a fee-for-service system [26–28]. Established by the NHIS, the NHIS-NSC database 2.2 is a longitudinal dataset containing a systemically sampled cohort of 2.2% of the entire population [26, 29]. It includes a cohort of 1 025 340 individuals randomly selected from 47 851 928 individuals in the 2002 NHID [26]. This cohort was followed for 17 years from 2002 to 2019, with annual updates to include newborns and compensate for the annual decrease of cohort size due to eligibility disqualifications (eg, death, emigration) to maintain the sample size [26, 29]. The dataset provides extensive information on healthcare resource utilization, procedures, prescription drugs, diagnostic codes, and personal details, aiding public health researchers and policy makers [26].

The number of patients with CCs was recalculated and evaluated each year, as additional patients were added each year, owing to the chronic status of these conditions. The cohort entry date (CED) was defined as the date when the individual met 1 of the following inclusion criteria: (*i*) the date of diagnosis for each CC based on the *International Classification of Diseases, 10th Revision* (*ICD-10*) diagnosis codes (Supplementary Table 1) [30]; or (*ii*) the date the patient turned 18 years of age, whichever occurred earlier. The earliest CED was 1 January 2010. Each patient was followed from CED until the end of the study period, or death, whichever occurred first. Patients with a prior history of HZ or related complications within 365 days before the CED in the NHIS-NSC database were excluded to ensure that HZ cases captured were incident cases.

Incident HZ patients were identified as those who had received an HZ or HZ-related complications diagnosis (*ICD-10* code B02) in hospital, outpatient, or emergency department settings and had received corresponding antiviral medication (eg, acyclovir, valacyclovir, or famciclovir) within ±7 days from the date of diagnosis of HZ during the follow-up period. CCs included were diabetes, chronic obstructive pulmonary disease (COPD), asthma, chronic kidney disease (CKD), and chronic liver disease (CLD). Complications of HZ infections (eg, cutaneous [including soft skin tissue infections], disseminated, ocular, neurologic, and other complications) were defined at presentation within 30 days after the initial date of HZ diagnosis at any setting. Patients with HZ who had ≥1 claim of *ICD-10* code B02.9 after the date of HZ diagnosis and no other HZ complications were categorized as zoster without complication, while those with ≥1 claim of *ICD-10* code B02.8 within 30 days after the date of HZ diagnosis were categorized as other. The full list of *ICD-10* diagnosis codes used for the diagnosis of HZ complication is described in Supplementary Table 1.

### HZ Incidence

Average annual IRs and IRRs (per 1000 persons) with associated 95% confidence intervals (CIs) were calculated in individuals with or without CCs during the study period. These were then stratified by age group (18–29, 30–39, 40–49, 50–59, 60–64, 65–69, 70–79, ≥80 years), sex, complications, and CCs.

Annual IRs of HZ were calculated as the number of HZ patients divided by the number of individuals aged ≥18 years, derived from NHIS-NSC on an annual basis. Average annual IRs for HZ in individuals with and without CCs were calculated based on annual IRs during the study period.

IRRs were used to assess the relative risk of HZ in individuals with CCs and were calculated by comparing the average annual IRs of HZ in the individuals with CCs to those without. Poisson regression and logarithm of the population size were used as offset terms.

## RESULTS

**Figure 1.**
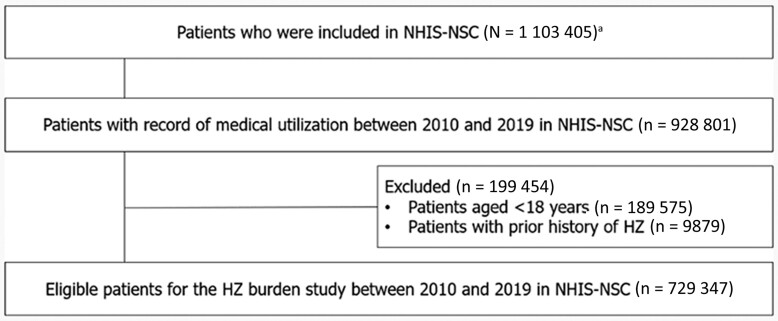
Inclusion and exclusion criteria of patients identified from the National Health Insurance Service National Sample Cohort database for this study. ^a^This data source is a longitudinal dataset containing a systematically sampled cohort of 2.2% of the nation's population (see Methods). Abbreviations: HZ, herpes zoster; NHIS-NSC, National Health Insurance Service National Sample Cohort.

Among the 1 103 405 individuals included in the NHIS-NSC database, a total of 729 347 individuals aged ≥18 years with a record of medical utilization from 2010 to 2019 in NHIS-NSC and without a prior history of HZ were considered eligible for inclusion in the study (Figure 1).

An average of 3323 HZ cases per year were identified in patients in the non-CC population (Table 1). Among the patients with specific CCs, the average number of HZ cases per year were 3203 for patients with asthma, 2697 for COPD, 2211 for diabetes, 380 for CLD, and 133 for CKD. The average annual HZ IR (95% CI; per 1000 persons) in the non-CC population was 9.67 (8.76–10.58). Among patients with specific CCs, HZ IRs were highest in patients with diabetes (15.96 [15.64–16.28]), followed by COPD (15.37 [14.89–15.85]), CKD (15.17 [13.46–16.88]), asthma (14.80 [14.22–15.38]), and CLD (13.61 [12.93–14.29]). Subsequently, the HZ IRRs (95% CI) ranged from 1.41 (1.26–1.57) for CLD to 1.65 (1.50–1.82) for diabetes.

**Table 1.** Average Annual Herpes Zoster Incidence Rates and Incidence Rate Ratios by Chronic Condition, 2010–2019

Chronic Condition	Average No. of HZ Cases per Year	Incident HZ	
		IR (95% CI)^a,b,c^	IRR (95% CI)^a,b,d^
Non-CC population (reference group)	3323	9.67 (8.76–10.58)	1.00 (ref)
Diabetes	2211	15.96 (15.64–16.28)	1.65 (1.50–1.82)
COPD	2697	15.37 (14.89–15.85)	1.59 (1.44–1.76)
Asthma	3203	14.80 (14.22–15.38)	1.53 (1.38–1.70)
Chronic liver disease	380	13.61 (12.93–14.29)	1.41 (1.26–1.57)
Chronic kidney disease	133	15.17 (13.46–16.88)	1.57 (1.35–1.82)

Abbreviations: CC, chronic condition; CI, confidence interval; COPD, chronic obstructive pulmonary disease; HZ, herpes zoster; IR, incidence rate; IRR, incidence rate ratio.

^a^Per 1000 persons.

^b^Based on Poisson distribution.

^c^IR is defined as the average annual IR of HZ in each CC population, which is calculated as the number of initial HZ cases in each CC population in a given year divided by the total number of each CC population in a given year. The average annual IR of HZ in each CC population is calculated as the sum of annual IRs of HZ in each CC population across the study period divided by the number of years of study period.

^d^IRR of HZ in each CC population is calculated by the annual IR of HZ in each CC population divided by the annual IR of HZ in the non-CC population (ie, patients without any CCs).

The age-specific annual number of incident HZ cases were consistently the highest in patients aged 50–59 years across the CCs (Table 2). The average annual HZ IRs generally increased with age, typically peaking at 60–64 or 65–69 years of age before declining at 70–79 and ≥80 years of age. In most age groups, HZ IRs were higher in patients with CCs than the non-CC individuals. The highest age-specific HZ IR was in the age group 65–69 years for asthma (20.83 [19.80–21.87]) and CLD (17.9 [15.76–20.05]) and in the age group 60–64 years for COPD (20.43 [19.76–21.10]), diabetes (18.75 [18.31–19.18]), and CKD (19.31 [17.44–21.18]). Interestingly, the HZ IRRs for patients with diabetes, CLD, or CKD were mostly higher in younger patients aged <50 years than patients aged ≥50 years.

**Table 2.** Average Annual Herpes Zoster Incidence Rates and Incidence Rate Ratios With 95% Confidence Intervals for Patients With or Without Chronic Conditions, 2010–2019, by Age Group

Chronic Condition	Age Group, y							
	18–29	30–39	40–49	50–59	60–64	65–69	70–79	≥80
Non-CC population (reference group)								
Average number of HZ cases per year	556	636	807	805	197	134	153	34
IR (95% CI)^a,b,c^	6.20 (5.29–7.11)	7.78 (6.74–8.81)	10.33 (8.83–11.82)	14.52 (13.31–15.74)	14.93 (14.21–15.65)	14.76 (13.88–15.65)	13.14 (12.26–14.02)	7.58 (5.92–9.24)
IRR (95% CI)^a,b,d^	1.00 (ref)	1.00 (ref)	1.00 (ref)	1.00 (ref)	1.00 (ref)	1.00 (ref)	1.00 (ref)	1.00 (ref)
Diabetes								
Average number of HZ cases per year	32	101	278	627	321	310	451	92
IR (95% CI)^a,b,c^	8.61 (7.25–9.98)	10.66 (9.40–11.92)	12.93 (11.30–14.56)	17.33 (16.54–18.11)	18.75 (18.31–19.18)	18.71 (18.08–19.34)	16.75 (15.32–18.18)	10.80 (8.47–13.13)
IRR (95% CI)^a,b,d^	1.39 (1.12–1.73)	1.37 (1.15–1.64)	1.25 (1.03–1.52)	1.19 (1.08–1.31)	1.26 (1.19–1.32)	1.27 (1.18–1.36)	1.27 (1.14–1.42)	1.42 (1.04–1.95)
COPD								
Average number of HZ cases per year	155	272	418	702	312	286	445	106
IR (95% CI)^a,b,c^	7.99 (6.80–9.17)	10.62 (9.02–12.22)	13.87 (11.87–15.87)	19.50 (18.64–20.36)	20.43 (19.76–21.10)	19.31 (18.24–20.38)	17.13 (15.73–18.53)	10.85 (8.39–13.32)
IRR (95% CI)^a,b,d^	1.29 (1.04–1.59)	1.37 (1.12–1.67)	1.34 (1.09–1.65)	1.34 (1.22–1.48)	1.37 (1.29–1.45)	1.31 (1.21–1.42)	1.30 (1.17–1.45)	1.43 (1.04–1.97)
Asthma								
Average number of HZ cases per year	260	412	578	797	316	304	436	101
IR (95% CI)^a,b,c^	7.62 (6.49–8.74)	10.59 (9.10–12.07)	13.62 (11.69–15.56)	19.75 (19.17–20.34)	20.64 (19.96–21.32)	20.83 (19.80–21.87)	18.07 (16.49–19.65)	11.64 (9.02–14.26)
IRR (95% CI)^a,b,d^	1.23 (1.00–1.51)	1.36 (1.12–1.65)	1.32 (1.08–1.62)	1.36 (1.24–1.49)	1.38 (1.30–1.47)	1.41 (1.31–1.53)	1.37 (1.23–1.54)	1.54 (1.12–2.11)
Chronic liver disease								
Average number of HZ cases per year	16	35	69	117	45	42	48	8
IR (95% CI)^a,b,c^	7.89 (6.61–9.17)	9.54 (8.48–10.59)	11.96 (10.00–13.92)	15.66 (14.42–16.90)	16.91 (14.83–18.99)	17.9 (15.76–20.05)	15.37 (13.56–17.17)	9.28 (7.13–11.43)
IRR (95% CI)^a,b,d^	1.27 (1.02–1.59)	1.23 (1.03–1.46)	1.16 (0.93–1.44)	1.08 (0.96–1.21)	1.13 (0.99–1.29)	1.21 (1.06–1.39)	1.17 (1.02–1.34)	1.22 (0.88–1.69)
Chronic kidney disease								
Average number of HZ cases per year	3	6	13	31	18	19	34	9
IR (95% CI)^a,b,c^	9.60 (5.85–13.36)	10.90 (8.30–13.50)	12.75 (10.12–15.38)	17.70 (15.84–19.57)	19.31 (17.44–21.18)	16.95 (15.14–18.76)	15.22 (12.79–17.66)	11.50 (4.90–18.10)
IRR (95% CI)^a,b,d^	1.55 (1.00–2.40)	1.40 (1.06–1.85)	1.23 (0.96–1.59)	1.22 (1.07–1.40)	1.29 (1.16–1.44)	1.15 (1.02–1.30)	1.16 (0.97–1.38)	1.52 (0.76–3.03)

Abbreviations: CC, chronic condition; CI, confidence interval; COPD, chronic obstructive pulmonary disease; HZ, herpes zoster; IR, incidence rate; IRR, incidence rate ratio.

^a^Per 1000 persons.

^b^Based on Poisson distribution.

^c^IR is defined as the average annual IR of HZ in each CC population, which is calculated as the number of initial HZ cases in each CC population in a given year divided by the total number of each CC population in a given year. The average annual IR of HZ in each CC population is calculated as the sum of annual IRs of HZ in each CC population across the study period divided by the number of years of study period.

^d^IRR of HZ in each CC population is calculated by the annual IR of HZ in each CC population divided by the annual IR of HZ in the non-CC population (ie, patients without any CCs).

The sex-specific average annual numbers of HZ cases were numerically higher among females compared to males across CCs, with the exception of CKD (Table 3). The same trend was observed for the corresponding sex-specific average annual HZ IRs. In contrast, the HZ IRRs were numerically higher for males than females for most CCs, except CLD, for which similar HZ IRRs (95% CI) were reported for females (1.46 [1.31–1.63]) and males (1.41 [1.25–1.59]). The highest IRR of 1.71 (1.48–1.96) was reported for male patients with CKD, although no statistical differences in IRRs were observed between males and females across all CCs.

**Table 3.** Average Annual Herpes Zoster Incidence Rates and Incidence Rate Ratios With 95% Confidence Intervals for Patients With or Without Chronic Conditions, 2010–2019, by Sex

Chronic Condition	Sex	
	Male	Female
Non-CC population (reference group)		
Average number of HZ cases per year	1397	1927
IR (95% CI)^a,b,c^	8.12 (7.31–8.92)	11.23 (10.17–12.28)
IRR (95% CI)^a,b,d^	1.00 (ref)	1.00 (ref)
Diabetes		
Average number of HZ cases per year	919	1292
IR (95% CI)^a,b,c^	13.49 (13.20–13.79)	18.32 (17.81–18.83)
IRR (95% CI)^a,b,d^	1.66 (1.50–1.84)	1.63 (1.48–1.80)
COPD		
Average number of HZ cases per year	1025	1672
IR (95% CI)^a,b,c^	13.23 (12.76–13.70)	17.07 (16.52–17.61)
IRR (95% CI)^a,b,d^	1.63 (1.47–1.81)	1.52 (1.38–1.68)
Asthma		
Average number of HZ cases per year	1051	2152
IR (95% CI)^a,b,c^	12.46 (11.91–13.01)	16.26 (15.59–16.92)
IRR (95% CI)^a,b,d^	1.54 (1.38–1.71)	1.45 (1.31–1.60)
Chronic liver disease		
Average number of HZ cases per year	181	198
IR (95% CI)^a,b,c^	11.45 (10.72–12.17)	16.43 (15.50–17.37)
IRR (95% CI)^a,b,d^	1.41 (1.25–1.59)	1.46 (1.31–1.63)
Chronic kidney disease		
Average number of HZ cases per year	70	62
IR (95% CI)^a,b,c^	13.84 (12.48–15.19)	16.77 (14.16–19.38)
IRR (95% CI)^a,b,d^	1.71 (1.48–1.96)	1.49 (1.24–1.79)

Abbreviations: CC, chronic condition; CI, confidence interval; COPD, chronic obstructive pulmonary disease; HZ, herpes zoster; IR, incidence rate; IRR, incidence rate ratio.

^a^Per 1000 persons.

^b^Based on Poisson distribution.

^c^IR is defined as the average annual IR of HZ in each CC population, which is calculated as the number of initial HZ cases in each CC population in a given year divided by the total number of each CC population in a given year. The average annual IR of HZ in each CC population is calculated as the sum of annual IRs of HZ in each CC population across the study period divided by the number of years of study period.

^d^IRR of HZ in each CC population is calculated by the annual IR of HZ in each CC population divided by the annual IR of HZ in the non-CC population (ie, patients without any CCs).

We also estimated the incidence of HZ with various complications (Table 4). The average annual numbers of cases were highest for HZ without complications, followed by HZ with other, cutaneous, ocular, neurologic (non-PHN) complications, PHN, and disseminated HZ in both CC patients and non-CC individuals, although the trend varied slightly by CC. A similar trend was seen for the average annual IR of HZ with the different complications among the CC and non-CC populations, while the IRRs were highest for HZ with neurologic (non-PHN) complications and lowest for HZ with no complications, and the trend varied by CC. The highest IRR of 4.11 (95% CI, 3.24–5.22) was seen for the IR of HZ with neurologic complications in patients with CKD.

**Table 4.** Average Annual Herpes Zoster Incidence Rates and Incidence Rate Ratios With 95% Confidence Intervals for Patients With or Without Chronic Conditions, 2010–2019, by HZ Complication

Chronic Condition	No Complications	Complications					
		Cutaneous	Disseminated	Ocular	Neurologic	PHN	Other
Non-CC population (reference group)							
Average number of HZ cases per year	1730	253	33	155	92	72	674
IR (95% CI)^a,b,c^	5.56 (5.10–6.03)	0.83 (0.66–1.00)	0.06 (0.05–0.07)	0.47 (0.37–0.57)	0.30 (0.26–0.34)	0.05 (0.05–0.06)	1.55 (1.51–1.59)
IRR (95% CI)^a,b,d^	1.00 (ref)	1.00 (ref)	1.00 (ref)	1.00 (ref)	1.00 (ref)	1.00 (ref)	1.00 (ref)
Diabetes							
Average number of HZ cases per year	1127	201	17	218	142	18	322
IR (95% CI)^a,b,c^	8.22 (7.84–8.59)	1.41 (1.23–1.58)	0.13 (0.11–0.14)	1.52 (1.32–1.72)	1.00 (0.91–1.10)	0.13 (0.10–0.17)	2.36 (2.21–2.51)
IRR (95% CI)^a,b,d^	1.48 (1.34–1.63)	1.70 (1.33–2.17)	2.05 (1.62–2.59)	3.23 (2.50–4.18)	3.37 (2.83–4.01)	2.48 (1.86–3.30)	1.52 (1.42–1.63)
COPD							
Average number of HZ cases per year	1388	241	20	226	156	22	410
IR (95% CI)^a,b,c^	7.96 (7.74–8.17)	1.53 (1.32–1.73)	0.12 (0.09–0.14)	1.26 (1.10–1.42)	0.88 (0.79–0.96)	0.13 (0.12–0.14)	2.38 (2.25–2.51)
IRR (95% CI)^a,b,d^	1.28 (1.01–1.63)	1.85 (1.44–2.37)	1.89 (1.44–2.48)	2.67 (2.07–3.45)	2.94 (2.48–3.49)	2.36 (1.95–2.84)	1.39 (1.11–1.75)
Asthma							
Average number of HZ cases per year	1675	332	20	261	171	21	483
IR (95% CI)^a,b,c^	7.79 (7.62–7.96)	1.48 (1.23–1.73)	0.10 (0.08–0.11)	1.18 (1.01–1.34)	0.78 (0.69–0.86)	0.10 (0.08–0.11)	2.26 (2.17–2.36)
IRR (95% CI)^a,b,d^	1.40 (1.28–1.53)	1.79 (1.37–2.35)	1.57 (1.20–2.05)	2.50 (1.92–3.25)	2.61 (2.17–3.13)	1.76 (1.41–2.20)	1.46 (1.39–1.54)
Chronic liver disease							
Average number of HZ cases per year	199	36	3	29	21	2	58
IR (95% CI)^a,b,c^	7.18 (6.82–7.55)	1.26 (1.06–1.46)	0.11 (0.06–0.15)	0.99 (0.79–1.20)	0.75 (0.58–0.92)	0.08 (0.05–0.11)	2.13 (1.94–2.32)
IRR (95% CI)^a,b,d^	1.29 (1.17–1.42)	1.52 (1.17–1.98)	1.74 (1.05–2.89)	2.11 (1.56–2.86)	2.51 (1.91–3.29)	1.42 (0.92–2.17)	1.37 (1.25–1.51)
Chronic kidney disease							
Average number of HZ cases per year	65	12	1	14	12	1	18
IR (95% CI)^a,b,c^	7.43 (6.32–8.54)	1.34 (0.98–1.71)	0.12 (0.06–0.19)	1.53 (1.33–1.72)	1.23 (1.00–1.46)	0.12 (0.06–0.17)	2.25 (1.71–2.78)
IRR (95% CI)^a,b,d^	1.34 (1.13–1.59)	1.63 (1.15–2.30)	2.02 (1.12–3.65)	3.24 (2.51–4.20)	4.11 (3.24–5.22)	2.18 (1.29–3.70)	1.45 (1.14–1.85)

Abbreviations: CC, chronic condition; CI, confidence interval; COPD, chronic obstructive pulmonary disease; HZ, herpes zoster; IR, incidence rate; IRR, incidence rate ratio; PHN, postherpetic neuralgia.

^a^Per 1000 persons.

^b^Based on Poisson distribution.

^c^IR is defined as the average annual IR of HZ in each CC population, which is calculated as the number of initial HZ cases in each CC population in a given year divided by the total number of each CC population in a given year. The average annual IR of HZ in each CC population is calculated as the sum of annual IRs of HZ in each CC population across the study period divided by the number of years of study period.

^d^IRR of HZ in each CC population is calculated by the annual IR of HZ in each CC population divided by the annual IR of HZ in the non-CC population (ie, patients without any CCs).

## DISCUSSION

In this study, we estimated the average annual HZ IRs (overall and by complications) and IRRs among adults aged ≥18 years with or without 5 CCs (diabetes, COPD, asthma, CKD, and CLD) in the ROK. Compared to the non-CC population, patients with CCs had more than 40% higher risk of HZ. A similar trend was observed for the IR of HZ with different complications as well as when the overall HZ IRs were stratified by age, sex, and type of CC.

While it has been well established that immunocompromised adults have a higher incidence of HZ and HZ-associated healthcare burden compared to their immunocompetent counterparts [31, 32], the risk of HZ in adults with underlying CCs has not been well documented in the ROK. The results reported in this study are consistent with findings from studies of CC patients in other countries. For example, in a German study, patients with asthma, chronic heart failure, COPD, depression, and rheumatoid arthritis had an approximately 30% higher risk of developing HZ compared to those without any underlying conditions [22]. Similarly, a Japanese study reported higher HZ IRs associated with specific CCs (eg, asthma, chronic hepatitis, cirrhosis, COPD), where HZ IRs in individuals with CCs ranged from 5.40 to 12.90 per 1000 person-years, compared to 4.92 per 1000 person-years in all individuals aged ≥18 years in the study [24].

As expected, we found that HZ incidence and risk were higher among adults ≥50 years of age regardless of preexisting CCs [8, 33]. Across CCs, variations were observed in the age group with the highest HZ risk. In the non-CC population—as well as patients with diabetes, COPD, or CKD—HZ IRs were highest among those 60–64 years of age, while for asthma or CLD, patients 65–69 years showed the highest HZ IR. Noteworthily, higher IRRs were observed in the younger (<50 years) age group compared with the age group ≥50 years for diabetes, CLD, and CKD. With the likelihood of having multiple comorbidities higher with increased age [34], these “younger” patients were less likely than their older peers to have multiple comorbidities, suggesting that the specific CC was more likely associated with the increased risk of HZ, even though they were below the commonly accepted age threshold of 50 years for increased HZ risk. These findings highlight the urgency of HZ prevention in younger patients with these CCs.

Current prevention strategies for HZ in the ROK may not be comprehensive [35, 36]. For the prevention of VZV infection, a single dose of varicella vaccine is provided by the National Immunization Program (NIP) for infants 12–15 months of age. Conversely, in many other countries, a booster dose of varicella vaccine is also offered in the NIP. For example, in the United States, universal varicella vaccination is implemented with a 2-dose schedule at 12–15 months of age and 4–6 years of age [36, 37]. For the prevention of VZV reactivation, the Korean adult immunization guidelines (2014) recommended HZ vaccination for adults aged ≥60 years, and adults aged 50–59 years depending on individual health conditions [38]. However, no public funding is provided and HZ vaccination needs to be self-paid. As a result, the HZ vaccine coverage among adults ≥50 years of age has been low, with an estimated coverage of 9.4% in 2015 [7]. The NHIS-NSC database used in this study does not include information on HZ vaccination status [26], and current data on HZ vaccination coverage are not available. In recent years, a number of global medical organizations developed HZ vaccination guidelines for patients with CCs (eg, COPD, diabetes) [39–41], and the recombinant zoster vaccine (RZV) was launched in ROK in December 2022 in addition to the previously available live-attenuated zoster vaccine (LZV) [42]. Accordingly, the 2023 update of the Korean adult immunization guidelines recommend vaccination with RZV for adults aged ≥50 years (LZV may be administered instead of RZV), and RZV vaccination is recommended for severely immunocompromised adults aged ≥18 years [43]. Importantly, alongside these developments in HZ vaccination options and recommendations, there is a growing awareness about healthy aging and recognition of the value of adult immunization. Moreover, given the continuous increase in the prevalence of CCs in the ROK due to its aging population, there is a clear need to reevaluate the HZ prevention strategy for the Korean population, especially for individuals at increased risk of HZ, such as adults with CCs [35].

One important aspect of the disease burden associated with HZ that remains to be fully understood is the impact of HZ on patients’ health conditions, especially for individuals with underlying comorbidities, such as the onset of new disease or events following HZ [44–46], worsening of patients’ underlying conditions [47], and increased use of medications and healthcare resources [23]. The present study focused on quantifying the risk of HZ in patients with selected CCs, leaving room for future studies to further explore the downstream effect of HZ on patients with CCs for more comprehensive understanding of HZ-associated burden in these patient groups.

When interpreting the findings of this study, limitations on the use of administrative claims data need to be considered. As medical claims data are collected mainly for reimbursement purposes, rather than research, there are some inherent limitations such as incomplete, inaccurate, or missing data. Specifically, as this study relied on the *ICD-10* diagnostic codes for measuring exposure, outcome, and covariate status from the procured data, there is a possibility for miscoding or misdiagnosis, which would consequently lead to under- or overestimation of the true rate of variables assessed. Information on nonreimbursed procedures and prescription medications and over-the-counter drugs were not captured and may also affect the estimates presented here. Confounding factors that cannot be assessed from the medical claims database (eg, environmental and lifestyle factors, markers of clinical severity, functional status, degree and potential risks of immunosuppression, and frailty) may affect the results. Furthermore, not all HZ patients receive antiviral medication treatment. One study conducted in the ROK reported that 67.6%, 67.9%, and 21.3% of HZ patients were prescribed antivirals, nonnarcotics, and anti-epileptics [48]. As such, the definition of incident HZ cases used in this study may artificially reduce the incidence of HZ.

The analysis was done for 5 preselected CCs, so results may not be generalizable to other CCs. Moreover, as this study did not differentiate between patients with a single CC and those with multiple CCs, it is challenging to draw conclusions regarding the risk of HZ associated with a specific CC. Nonetheless, the study provided new evidence regarding the increased risk of HZ among patients with these CCs, such data taken within the context of the rapidly aging population and increasing prevalence of CCs in the ROK, highlighting the need for and urgency of HZ prevention.

## CONCLUSIONS

In this nationwide database study in the ROK, patients with selected CCs showed an increased risk of HZ compared with those without underlying CCs. Findings of this study reiterate the importance of HZ prevention for individuals with underlying comorbidities in the ROK, which may contribute to the healthy aging of the Korean population.

## Supplementary Material

ofae535_Supplementary_Data

## Supplementary Data

Supplementary materials are available at *Open Forum Infectious Diseases* online. Consisting of data provided by the authors to benefit the reader, the posted materials are not copyedited and are the sole responsibility of the authors, so questions or comments should be addressed to the corresponding author.

## Notes

***Acknowledgments.*** The authors thank Costello Medical for editorial assistance and publication coordination, on behalf of GSK, and acknowledge Loveena Sharma, Costello Medical, Singapore, for medical writing and editorial assistance based on authors’ input and direction.

***Author contributions.*** All authors made substantial contributions to study conception, study design, data analysis and interpretation, drafting of the article or revising it critically for important intellectual content, and final approval of the manuscript.

***Patient consent.*** This study does not include factors necessitating patient consent.

***Data sharing.*** According to the National Health Insurance Service National Sample Cohort database policy, source data cannot be shared.

***Disclaimer.*** Support for third-party writing assistance for this article, provided by Loveena Sharma, Costello Medical, Singapore, was funded by GSK in accordance with Good Publication Practice 2022 guidelines (https://www.ismpp.org/gpp-2022).

***Financial support*.** This study was sponsored by GSK (study identifier VEO-000415).

## References

[1] Gnann JW Jr, Whitley RJ. Clinical practice. Herpes zoster. N Engl J Med 2002; 347:340–6. 12151472 10.1056/NEJMcp013211

[2] Arvin A . Aging, immunity, and the varicella-zoster virus. N Engl J Med 2005; 352:2266–7. 15930416 10.1056/NEJMp058091

[3] Centers for Disease Control and Prevention . Clinical features of chickenpox (varicella). 2024. Available at: https://www.cdc.gov/chickenpox/hcp/clinical-signs/index.html. Accessed 23 September 2024.

[4] Tseng HF, Smith N, Harpaz R, Bialek SR, Sy LS, Jacobsen SJ. Herpes zoster vaccine in older adults and the risk of subsequent herpes zoster disease. JAMA 2011; 305:160–6. 21224457 10.1001/jama.2010.1983

[5] Centers for Disease Control and Prevention . Prevention of herpes zoster recommendations of the Advisory Committee on Immunization Practices (ACIP). **2008**. Available at: https://www.cdc.gov/mmwr/preview/mmwrhtml/rr5705a1.htm. Accessed 17 April 2024. 18528318

[6] Centers for Disease Control and Prevention . Clinical features of shingles (herpes zoster). 2024. Available at: https://www.cdc.gov/shingles/hcp/clinical-signs/index.html#cdc_hcp_clinical_complications-complications. Accessed 23 September 2024.

[7] Choi JK, Park SH, Park S, et al Trends in varicella and herpes zoster epidemiology before and after the implementation of universal one-dose varicella vaccination over one decade in South Korea, 2003–2015. Hum Vaccin Immunother 2019; 15:2554–60. 31008679 10.1080/21645515.2019.1603985 PMC6930048

[8] Kim YJ, Lee CN, Lim CY, Jeon WS, Park YM. Population-based study of the epidemiology of herpes zoster in Korea. J Korean Med Sci 2014; 29:1706–10. 25469074 10.3346/jkms.2014.29.12.1706 PMC4248595

[9] Grote V, von Kries R, Rosenfeld E, Belohradsky BH, Liese J. Immunocompetent children account for the majority of complications in childhood herpes zoster. J Infect Dis 2007; 196:1455–8. 18008223 10.1086/522631

[10] Yawn BP, Saddier P, Wollan PC, et al A population-based study of the incidence and complication rates of herpes zoster before zoster vaccine introduction. Mayo Clin Proc 2007; 82:1341–9. 17976353 10.4065/82.11.1341

[11] Joesoef RM, Harpaz R, Leung J, Bialek SR. Chronic medical conditions as risk factors for herpes zoster. Mayo Clin Proc 2012; 87:961–7. 23036671 10.1016/j.mayocp.2012.05.021 PMC3538398

[12] Bagatini MD, Cardoso AM, Reschke CR, Carvalho FB. Immune system and chronic diseases 2018. J Immunol Res 2018; 2018:8653572. 30474045 10.1155/2018/8653572 PMC6220381

[13] Lai SW, Liu CS, Kuo YH, Lin CL, Hwang BF, Liao KF. The incidence of herpes zoster in patients with diabetes mellitus: a meta-analysis of cohort studies. Medicine (Baltimore) 2021; 100:e25292. 33879659 10.1097/MD.0000000000025292 PMC8078473

[14] Yang Y-W, Chen Y-H, Wang K-H, Wang C-Y, Lin H-W. Risk of herpes zoster among patients with chronic obstructive pulmonary disease: a population-based study. CMAJ 2011; 183:E275–80. 21343261 10.1503/cmaj.101137 PMC3060212

[15] Marra F, Parhar K, Huang B, Vadlamudi N. Risk factors for herpes zoster infection: a meta-analysis. Open Forum Infect Dis 2020; 7:ofaa005. 32010734 10.1093/ofid/ofaa005 PMC6984676

[16] Forbes HJ, Bhaskaran K, Thomas SL, Smeeth L, Clayton T, Langan SM. Quantification of risk factors for herpes zoster: population based case-control study. BMJ 2014; 348:g2911. 25134101 10.1136/bmj.g2911 PMC4019782

[17] Shrestha AB, Umar TP, Mohammed YA, et al Association of asthma and herpes zoster, the role of vaccination: a literature review. Immun Inflamm Dis 2022; 10:e718. 36301037 10.1002/iid3.718 PMC9552974

[18] Peng Y-H, Fang H-Y, Wu B-R, et al Adult asthma is associated with an increased risk of herpes zoster: a population-based cohort study. J Asthma 2017; 54:250–7. 27410999 10.1080/02770903.2016.1211142

[19] Jeon D, Kim YJ, Kim S, et al Liver cirrhosis increases the risk of herpes zoster: a nationwide population-based cohort study. Am J Gastroenterol 2023; 118:1592–600. 36746415 10.14309/ajg.0000000000002209

[20] Li Z, Wang Q, Ma J, et al Risk factors for herpes zoster in patients with chronic kidney disease: a case-control study. Vaccines (Basel) 2021; 9:963. 34579200 10.3390/vaccines9090963 PMC8473266

[21] Morena D, Lumbreras S, Rodríguez JM, et al Chronic respiratory diseases as a risk factor for herpes zoster infection. Arch Bronconeumol 2023; 59:797–804. 37734964 10.1016/j.arbres.2023.08.010

[22] Batram M, Witte J, Schwarz M, et al Burden of herpes zoster in adult patients with underlying conditions: analysis of German claims data, 2007–2018. Dermatol Ther (Heidelb) 2021; 11:1009–26. 33959878 10.1007/s13555-021-00535-7 PMC8163947

[23] Piazza MF, Paganino C, Amicizia D, et al The unknown health burden of herpes zoster hospitalizations: the effect on chronic disease course in adult patients ≥50 years. Vaccines (Basel) 2020; 8:20. 31936724 10.3390/vaccines8010020 PMC7157675

[24] Imafuku S, Matsuki T, Mizukami A, et al Burden of herpes zoster in the Japanese population with immunocompromised/chronic disease conditions: results from a cohort study claims database from 2005–2014. Dermatol Ther (Heidelb) 2019; 9:117–33. 30456446 10.1007/s13555-018-0268-8 PMC6380970

[25] Baek JY, Lee E, Jung HW, Jang IY. Geriatrics fact sheet in Korea 2021. Ann Geriatr Med Res 2021; 25:65–71. 34187140 10.4235/agmr.21.0063 PMC8272996

[26] Lee J, Lee JS, Park SH, Shin SA, Kim K. Cohort profile: the National Health Insurance Service–National Sample Cohort (NHIS-NSC), South Korea. Int J Epidemiol 2017; 46:e15. 26822938 10.1093/ije/dyv319

[27] Cheol Seong S, Kim YY, Khang YH, et al Data resource profile: the national health information database of the National Health Insurance Service in South Korea. Int J Epidemiol 2017; 46:799–800. 27794523 10.1093/ije/dyw253 PMC5837262

[28] National Health Insurance Service . 2023 booklet for the introduction of national health insurance system. **2023**. Available at: https://www.nhis.or.kr/english/wbheaa03500m01.do?mode=view&articleNo=10835244&article.offset=0&articleLimit=10. Accessed 11 July 2024.

[29] Park I . How to use health insurance data effectively for healthcare research. J Health Info Stat 2022; 47:S31–9.

[30] World Health Organization . International statistical classification of diseases and related health problems (10th ed). **2019**. Available at: https://icd.who.int/browse10/2019/en#/. Accessed 10 January 2024.

[31] Buchan SA, Daneman N, Wang J, et al Incidence of hospitalizations and emergency department visits for herpes zoster in immunocompromised and immunocompetent adults in Ontario, Canada, 2002–2016. Clin Infect Dis 2020; 71:22–9. 31436814 10.1093/cid/ciz769

[32] McKay SL, Guo A, Pergam SA, Dooling K. Herpes zoster risk in immunocompromised adults in the United States: a systematic review. Clin Infect Dis 2020; 71:e125–34. 31677266 10.1093/cid/ciz1090 PMC7195255

[33] Choi JK, Park SH, Park S, et al The changing epidemiology of herpes zoster over a decade in South Korea, 2006–2015. Vaccine 2019; 37:5153–60. 31377077 10.1016/j.vaccine.2019.07.086

[34] Salive ME . Multimorbidity in older adults. Epidemiol Rev 2013; 35:75–83. 23372025 10.1093/epirev/mxs009

[35] Choi WS . Adult immunization policy in Korea. Infect Chemother 2023; 55:317–21. 37794577 10.3947/ic.2023.0089 PMC10551718

[36] Oh SH, Choi EH, Shin SH, et al Varicella and varicella vaccination in South Korea. Clin Vaccine Immunol 2014; 21:762–8. 24671555 10.1128/CVI.00645-13 PMC4018876

[37] Centers for Disease Control and Prevention . Varicella vaccination information for healthcare professionals. **2021**. Available at: https://www.cdc.gov/vaccines/vpd/varicella/hcp/index.html. Accessed 11 July 2024.

[38] Choi WS, Choi JH, Kwon KT, et al Revised adult immunization guideline recommended by the Korean Society of Infectious Diseases, 2014. Infect Chemother 2015; 47:68–79. 25844267 10.3947/ic.2015.47.1.68 PMC4384453

[39] ElSayed NA, Aleppo G, Aroda VR, et al Comprehensive medical evaluation and assessment of comorbidities: standards of care in diabetes—2023. Diabetes Care 2023; 46(Suppl 1):S49–67. 36507651 10.2337/dc23-S004 PMC9810472

[40] International Diabetes Foundation . IDF Europe position paper on vaccination of people living with diabetes. **2021**. Available at: https://idf.org/europe/news/idf-europe-position-paper-on-vaccination-of-people-living-with-diabetes/. Accessed 11 July 2024.

[41] Global Initiative for Chronic Obstructive Lung Diseases . Global strategy for prevention, diagnosis and management of COPD: 2023 report. **2023**. Available at: https://goldcopd.org/2023-gold-report-2/. Accessed 11 July 2024.

[42] Lee H-S. GSK Korea launches game-changing shingles vaccine. **2022**. Available at: https://www.koreabiomed.com/news/articleView.html?idxno=20006. Accessed 11 July 2024.

[43] Choi WS, Song JY, Kwon KT, et al Recommendations for adult immunization by the Korean Society of Infectious Diseases, 2023: minor revisions to the 3rd edition. Infect Chemother 2024; 56:188–203. 38960738 10.3947/ic.2023.0072 PMC11224039

[44] Cha M-J, Seo H-M, Choi E-K, et al Increased risk of atrial fibrillation in the early period after herpes zoster infection: a nationwide population-based case-control study. J Korean Med Sci 2018; 33:e160. 29805341 10.3346/jkms.2018.33.e160 PMC5966375

[45] Kwon SU, Yun SC, Kim MC, et al Risk of stroke and transient ischaemic attack after herpes zoster. Clin Microbiol Infect 2016; 22:542–8. 26992774 10.1016/j.cmi.2016.03.003

[46] Wu PY, Lin CL, Sung FC, Chou TC, Lee YT. Increased risk of cardiovascular events in patients with herpes zoster: a population-based study. J Med Virol 2014; 86:772–7. 24482346 10.1002/jmv.23892

[47] Lin SY, Liu JH, Yeh HC, et al Association between herpes zoster and end stage renal disease entrance in chronic kidney disease patients: a population-based cohort study. Eur J Clin Microbiol Infect Dis 2014; 33:1809–15. 24838650 10.1007/s10096-014-2143-6

[48] Cheong C, Lee TJ. Prevalence and healthcare utilization of herpes zoster and postherpetic neuralgia in South Korea: disparity among patients with different immune statuses. Epidemiol Health 2014; 36:e2014012. 25119454 10.4178/epih/e2014012 PMC4153010

