# Supplementary material for: Burden of Herpes Zoster in Individuals With Chronic Conditions in the Republic of Korea: A Nationwide Population-Based Database Study
Source: Open Forum Infect Dis. 2024 Sep 18;11(10):ofae535. doi: 10.1093/ofid/ofae535 (PMC11443341; doi:10.1093/ofid/ofae535)
Supplement: ofae535_Supplementary_Data [file ofae535_supplementary_data.pdf]

SUPPLEMENTARY MATERIALS

Supplementary Table 1. CCs, HZ complications, and their respective ICD-10 codes

	**Definition/ICD-10 codes[a]**
**CCs**	
Diabetes	At least one inpatient or outpatient diagnosis<br>ICD-10: E11, E12, E13, E14
COPD	At least one inpatient or outpatient diagnosis<br>ICD-10: J41, J42, J43, J44
Asthma	At least one inpatient or outpatient diagnosis<br>ICD-10: J45
Depression	At least one inpatient or outpatient diagnosis<br>ICD-10: F32, F33
Chronic kidney disease	At least one inpatient or outpatient diagnosis<br>ICD-10: N01, N03, N052, N053, N054, N055, N056, N072, N073, N074, N18, N19, N25, Z49, Z940, Z992
Chronic liver disease	At least one inpatient or outpatient diagnosis<br>ICD-10: B18, K703, K74, K743, K744, K745, K756
**HZ complications**	
Cutaneous	At least one claim within 30 days after the initial date of herpes zoster diagnosis<br>ICD-10: L03.x, L089, M726, A400, A410, L88
Disseminated	At least one claim within 30 days after the initial date of herpes zoster diagnosis<br>ICD-10: B02.7 only
Ocular	At least one claim within 30 days after the initial date of herpes zoster diagnosis<br>ICD-10: H03.1, H131, H220, H209, H192, H16, H190, H150, H570, H440, H024, H54, H309, H358
Neurologic	At least one claim within 30 days after the initial date of herpes zoster diagnosis<br>ICD-10: G020, A879, G527, G510, H903, H904, G051, J986, G628, G373, G048
PHN	At least one claim between 90 and 365 days after initial date of herpes zoster diagnosis<br>ICD-10: B02.2 or G53.0
Other complications	At least one claim within 30 days after the date of herpes zoster diagnosis<br>ICD-10: B02.8

	**Definition/ICD-10 codes[a]**
No complications	At least one claim of ICD-10: B02.9 after the date of herpes zoster diagnosis and none of above<br>ICD-10: B02.9 and none of above

AID: autoimmune disease; **COPD:** chronic obstructive pulmonary disease; **HZ:** herpes zoster; **IC:** immunocompromised; **ICD-10:** International Classification of Diseases, 10th revision; **PHN:** postherpetic neuralgia. [a]History of cirrhosis required to have at least one inpatient or outpatient diagnosis (ICD-10: K703, K74, K743, K744, K745, K756).
